# Increasing the Service Life of Marine Transport Using Heat-Resistant Polymer Nanocomposites

**DOI:** 10.3390/ma17071503

**Published:** 2024-03-26

**Authors:** Oleksandr Sapronov, Andriy Buketov, Boksun Kim, Pavlo Vorobiov, Lyudmila Sapronova

**Affiliations:** 1 Department of Transport Technologies and Mechanical Engineering, Kherson State Maritime Academy, Ushakova Avenue, 20, 73003 Kherson, Ukraine; buketov@tntu.edu.ua (A.B.); vorobyov020291@gmail.com (P.V.); 2023sapronova2023@gmail.com (L.S.); 2 School of Engineering, Computing and Mathematics, University of Plymouth, Drake Circus, Plymouth PL4 8AA, UK; boksun.kim@plymouth.ac.uk

**Keywords:** nanodispersed condensed carbon, nanocomposite, activation thermal destruction, infrared spectroscopy

## Abstract

This paper presents the technological aspects of increasing the thermal stability of polymers, with epoxy binder used to form the polymer materials. Polyethylene polyamine was used to crosslink the epoxy binder. To ensure the thermal stability of the polymer, nanodispersed condensed carbon with a dispersion of 10–16 nm was used. The research into nanocomposites under the influence of elevated temperatures was carried out using the “Thermoscan-2” derivatograph. Complex studies of thermophysical properties were carried out, according to the results of which the optimal content of nanofiller (0.050 pts.wt.) was determined. At the same time, this particular polymer was characterized by the following properties: temperature of the beginning of mass loss—*T*_0_ = 624.9 K; final temperature of mass loss—*T_f_* = 718.7 K; relative mass loss—*ε_m_* = 60.3%. Research into the activation energy of thermal destruction was performed to determine the resistance to the destruction of chemical bonds. It was proved that the maximum value of activation energy (170.1 kJ/mol) is characterized by nanocomposites with a content of nanodispersed condensed carbon of 0.050 pts.wt., which indicates the thermal stability of the polymer.

## 1. Introduction

Thanks to their improved mechanical, physical, and chemical properties, reactive plastics are used as adhesive materials or multi-functional products in many industries [1,2,3,4,5,6,7,8,9]. In terms of corrosion resistance, reactive plastic polymers are enormously superior to metals and their alloys [10,11,12,13], so they can be used as protective coatings, for example, when operating parts of transport equipment such as the hull parts of ships. Under the influence of high temperatures and pressure, the indicators of polymers can ensure efficiency in different applications.

The research background of this study is that unfilled reaction–plastic polymer materials do not provide high thermal stability [14,15,16,17,18] and are characterized by one of the disadvantages that limits their use—a low temperature at thermal destruction [19,20,21,22]. In addition, in the works [14,15,16,17,18,19] the technology of forming composite materials with nano particles is presented, which includes complex technological modes; in particular, functionalization in a solvent, solvent removal, ultrasonic dispersion, grafting of surface-active groups, and vacuuming. That is, the forming technology becomes more complicated and, accordingly, the forming time and the cost of the polymer material increase.

Therefore, the main task of scientific research is to ensure the thermal stability of polymers by choosing a thermally stable additive, as well as simplifying the technology of polymer material formation.

Carbon nanofillers, such as nanotubes, fullerenes, nanocarbon black, and graphene, are used to improve strength characteristics and prevent thermal destruction or to increase the temperature at which the combustion process occurs [16,18,23,24,25,26]. The authors of [27,28,29,30,31,32,33] investigated the course of the process of thermal destruction of polymers. These provisions are consistent with the results of the research given in the work by authors [18,19,20,21,22,23,24,25,26,27]. Therefore, the use of chemical methods for influencing the processes of thermo-oxidative destruction will allow the structure of such materials to be changed by introducing active nanosized particles into the polymer matrix. Nano-dispersed condensed carbon is the starting product in the detonation synthesis of nanodiamonds, as well as fullerene black or fullerene soot, formed during the synthesis of fullerenes. However, if the characteristics of fullerene soot and fullerene black are inferior to fullerenes, then the nanodispersed condensed carbon is similar in characteristics to nanodiamonds. Therefore, the main reasons for the use of nanodispersed condensed carbon are its characteristics, in particular, the specific surface area of nanodispersed condensed carbon is 280–460 m^2^/g, while the specific surface area of nanodiamond is 270–330 m^2^/g; the pore volume of nanodispersed condensed carbon is 0.6–0.9 cm^3^/g, while that of nanodiamond is 0.6–1.1 cm^3^/g, which is quite close in absolute value.

In terms of morphology, microstructure, elemental composition, and reactivity, nanodispersed condensed carbon and nanodiamonds are close to each other. The main difference is that the crystal structure of nanodispersed condensed carbon is hexagonal, and that of nanodiamond is cubic. Thus, the highly deformed state of the crystal lattice nanodispersed condensed carbon is thermally stable. This creates interest in the development of thermostable epoxy composites. This, in turn, will ensure a change in the mechanism and speed of chemical destruction reactions, and, therefore, a change in the thermo-oxidative destruction by inhibiting these reactions.

In addition, it should be noted that the cost of nanodispersed condensed carbon is 40% lower than nanodiamonds, which affects the cost performance of the final product, and therefore the scale of production for polymer materials.

## 2. Materials and Methods

### 2.1. Materials

Epoxy oligomer ED-20 was used as a binder (ISO 18280:2010) [10,20], which was hardened with polyethylene polyamine (Technical Regulations TU 6-05-241-202-78; Technobudresurs, Kyiv, Ukraine).

Nanodispersed condensed carbon (NCC) (YongFeng Chemicals Company, Hefei, China) was obtained by detonation synthesis. The size of the nanoparticles amounted to d ¼ 10–16 nm. The starting temperature of NCC oxidation was 583–623 K.

### 2.2. Material-Forming Technology

To improve the degree of nanoparticle wetting (the main cause of polymer delamination), and, therefore, the interphase interaction of the polymer–nanoparticle system, epoxy composites are formed:

- preliminary dosing of oligomer, heated to a temperature of 353 ± 2 K and held for a time of 20 ± 0.1 min;
- dosage of nanodispersed condensed carbon;
- introduction of nanodispersed condensed carbon into the composition in the following ratio—50% of additive to the epoxy binder, 50% of additive to the PEPA hardener;
- mechanical combination epoxy oligomer and nanodispersed condensed carbon during—1 ± 0.1 min;
- ultrasonic treatment (UST)—1.5 ± 0.1 min;
- cooling the composition (273 K)—60 ± 5 min;
- mechanical combination of PEPA and nanodispersed condensed carbon during—1 ± 0.1 min;
- ultrasonic treatment (UST)—1.5 ± 0.1 min;
- combination of two compositions (ED-20 with nanodispersed condensed carbon + PEPA with nanodispersed condensed carbon) during the time—5 ± 0.1 min.

Then, the polymerization of polymers under the set conditions was carried out [25,26,27]: time of the formation polymers—12.0 ± 0.1 h (temperature—293 ± 2 K), heat treatment of polymers at the temperature—393 ± 2 K (during the time—2.0 ± 0.05 h), cooling to the temperature—293 ± 2 K.

### 2.3. Research Methods

The advantage of the proposed technology over existing solutions is a simplified forming technology, which provides for a reduction in costs for the production of new materials; it allows materials to be formed in the conditions of a ship, making it possible to transport and store such materials on the ship on the one hand, and to ensure high operational characteristics (compared to analogues) on the other hand.

The structural changes that occur when the developed polymer materials were heated were studied using methods of thermogravimetric (TGA) and differential thermal analysis (DTA), and derivatograph “Thermoscan-2” was applied [21,27,33]. Polymeric materials were studied at a range of temperatures Δ*T* = 298–873 K, using quartz crucibles for specimens with a volume of *V* = 0.5 cm^3^ [21]. The rate of increase in temperature was *υ* = 5 K/min; moreover, the reference substance was Al_2_O_3_ (*m* = 0.5 g) [27]. The error of determining the temperature was Δ*T* = ±1 K [33]. The accuracy of determining thermal effects was 3 J/g. The accuracy of determining the weight was—Δm = 0.02 g [21].

**Figure 1:**
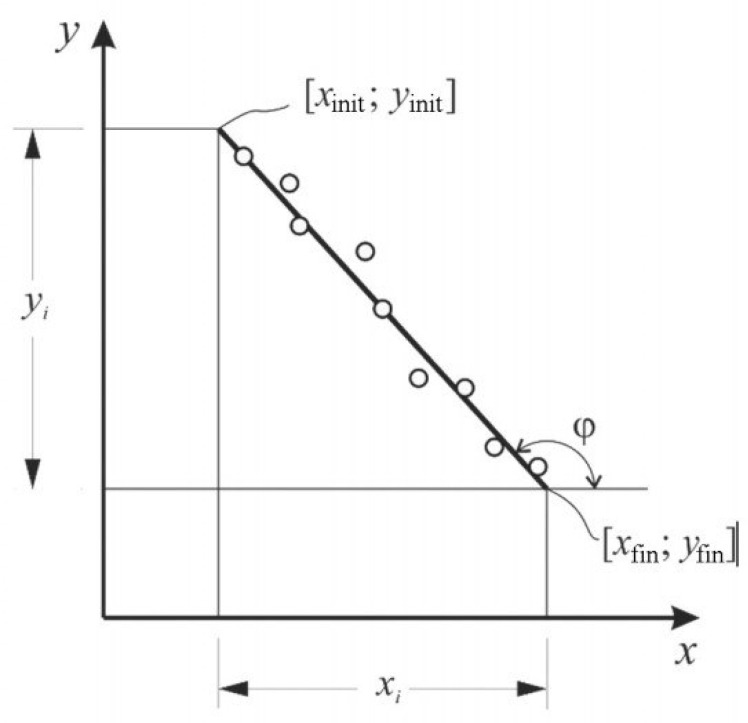
Graphical determination of activation energy.

The activation energy was determined by the Broido method [27,31]. The condition for using this method was the first order decomposition reaction, which applied to both thermosetting and thermoplastic polymers. The loss of mass of a substance is a process of the 1st order (*n* = 1), if the linear dependence of ln (100/(100 − Δm)) on the inverse temperature is 103/T, K^−1^. To determine the activation energy, a line was constructed—a line in which E must be expressed by the tangent of the angle of the logarithmic dependence Δm on the inverse temperature *T* (Figure 1). Energy activation of thermal destruction can be calculated by (1):

*E_a_* = −R·tg(φ) (1)

To graphically determine the activation energy of thermal destruction, the graph should have a straight line, the tangent of the angle of inclination φ of which makes it possible to calculate the activation energy *E_a_* (Figure 1) [27].

Then [31],

−tg(φ) = y_i_/ x_i_, (2)

*E* = R·y_i_/ x_i_, (3)

where x_i_= x_init_ − x_fin_—the length of the line along the abscissa; 

y_i_= y_init_ − y_fin_—the length of the line along the ordinate; 

[x_init_; y_init_] and [x_fin_; y_fin_]—coordinates of the beginning and end of the line, respectively.

With the use of the modern research method, IR spectral analysis, the mechanisms of the physical and chemical interaction of the binder with nanoadditives and their change during operation will be established [27]. The IR spectra were recorded on a spectrophotometer “IRAffinity-1” (Japan) in the field of wave numbers ν = 400–4000 cm^−1^ by a single-beam method in the reflected light. The wavelength scanning by wave numbers *λ*^−1^ = *ν* was performed on the diagram within 225 mm in the range of the selected frequencies. Wave numbers, the intensity of passage, the half-width, and the area of the absorption band were determined using a computer program called IRsolution. The error in determining the wave number was *ν* = ±0.01 cm^−1^, and the error in determining the peak location was *ν* = ±0.125 cm^−1^. The photometric accuracy was ± 0.2% in the case of a programmed control of a slit and the duration of integration was *t* = 10 s. The integration step was Δ*λ* = 4 cm^−1^. The IR spectral analysis of nanocomposites was performed with the optimal content of nanoparticles at different stages of thermal degradation. The material was crushed, dried at a temperature of *T* = 373 K ± 2 during *t* = 20 min, stirred in an agate mortar with the KBr powder, and then specimens were formed on a hydraulic press with a loading of *σ* = 20 MPa in the following proportions: study material—1 mg; KBr—300 mg [27].

## 3. Discussion

### 3.1. Thermogravimetric Analysis (TGA) of Composites Filled with a Nanodispersed Condensed Carbon

**Figure 2:**
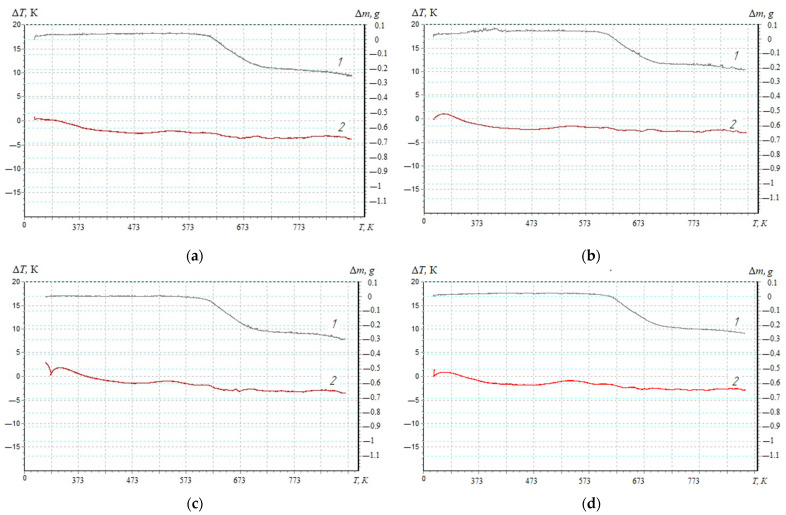
Thermal analysis of composite materials filled with a nanodispersed condensed carbon: (**a**) 0.025%; (**b**) 0.050 %; (**c**) 0.075%; (**d**) 0.100%; 1—thermogravimetric (1) analysis; 2—differential–thermal (2) analysis.

The thermal stability of polymers is one of the important characteristics that allows such materials to be used in different temperature ranges [22,23,24]. At the same time, thermal analysis is a method for determining the thermophysical properties of polymers and studying their temperature transitions. Therefore, previously in this work, the heat resistance of reactive plastic polymer materials (temperature range—303–873 K) was determined [21,27]. TGA analysis made it possible to establish the absence of mass loss for the developed nanocomposite materials in the temperature range—303–624 K (Figure 2, Table 1).

Structural changes in the unfilled polymer matrix occurred at T_0_ = 587 K [27]. Changing the content of nanofiller in the epoxy binder (0.025–0.100 pts.wt.) allowed the initial temperature of mass loss to be shifted by 37.0–47.9 K to the area of high temperatures.

Polymers filled with nanodispersed condensed carbon (0.050 pts.wt.) were characterized by the highest temperature of the beginning of mass loss (among the investigated nanocomposites) (Table 1), which makes it possible to claim inhibition of thermo-oxidative destruction reactions by limiting the mobility of segments of the polymer network and the main chain. The end of the destruction process of the developed composites was observed in the temperature—712–718 K. At the same time, the difference in the mass loss of the unfilled matrix (80.7%) [27] and the filled composites (63–70%) indicated the presence of a nanoadditive that underwent thermal transformations under the influence of a higher temperature.

### 3.2. Calculation of the Activation Energy of Thermal Destruction of Composite Materials Filled with a Nanodispersed Condensed Carbon

**Figure 3:**
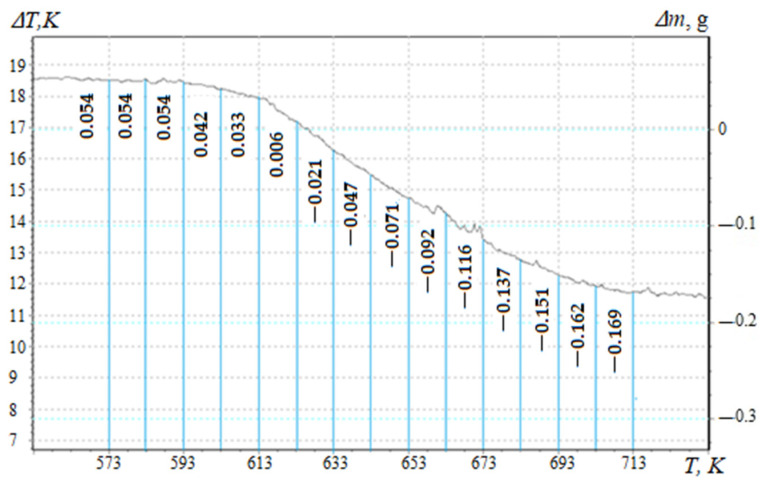
Mass loss of a composite material filled with nanodispersed condensed carbon (0.050 pts.wt.).

To assess the degree of crosslinking of composite materials filled with nanodispersed condensed carbon, the activation energy of thermal destruction is calculated according to the Broido method [27,31,33]. TGA curves (Figure 2, Curve 1) are used to mathematically calculate the activation energy, which are analyzed in the temperature range ∆T = 573–713 K (Figure 3), which corresponds to the loss of mass of polymers T_5-90_%, K. Previously, the mass loss of materials with an interval of ∆T = 10 K (Figure 3) for a composite filled with NCC (0.050 pts.wt.) was determined. Similarly, research was conducted for composites with different contents of nanofiller (Table 2, Table 3 and Table 4).

The mass value of the studied composite material is calculated as a percentage using Equation (4):

(4) $$\left(100-∆m\right)\%=\left(100-\left(\frac{m_{in}+∆m}{∆m}\cdot 100\right)\right)\%,$$

where m_in_—sample mass at the temperature (T_1_ = 573 K = const); 

∆m—mass of the polymer as the temperature increases.

The mass of the composite material at the initial temperature is taken as 100%. Table 2 shows the results of processing the TGA curves and the parameters necessary to calculate the energy activation of composites filled with NCC.

The activation energy was determined by the Broido method, according to works [25,29,31]:

(5) $$\ln\left(ln\frac{100}{100-∆m}\right)=-\frac{E}{R}\;\cdot \;\frac{1}{T}+const$$

The results of the calculations of the value of the double logarithm of the change in the mass of the samples are given in Table 3.

Knowing the mass loss (Δm) of composites filled with nanodispersed condensed carbon at temperature T, a straight line is graphically constructed, in which E is determined by the tangent of the slope angle of the logarithmic dependence of Δm on the inverse temperature T.

**Figure 4:**
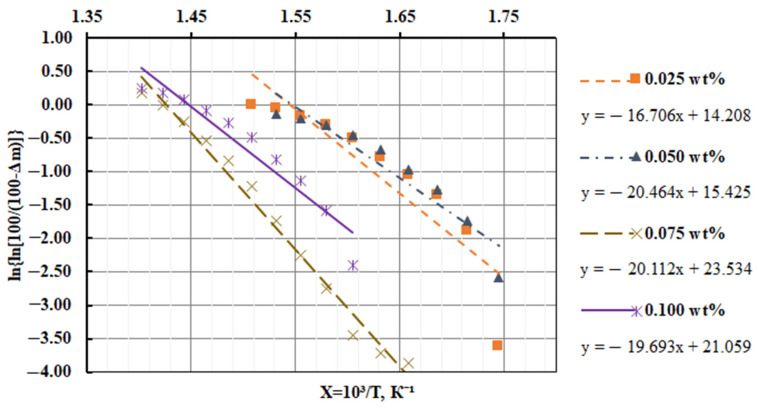
Graphical dependence of the epoxy composite materials filled with a nanodispersed condensed carbon destruction rate on the inverse temperature.

Destruction activation energy is found by Equation (1). Figure 4 shows the graphical dependences of the rate of destruction on the inverse temperature, and Table 4 shows the analytical results of the graphical determination of the activation energy of the developed CM.

Based on the calculations, it was found that for the thermal destruction of the composite filled with NCC (0.050 pts.wt.), the highest thermal energy (E_a_ = 170.1 kJ/mol) was required (Table 4).

### 3.3. Differential Thermal Analysis (DTA) of Composites Filled with a Nanodispersed Condensed Carbon

**Figure 5:**
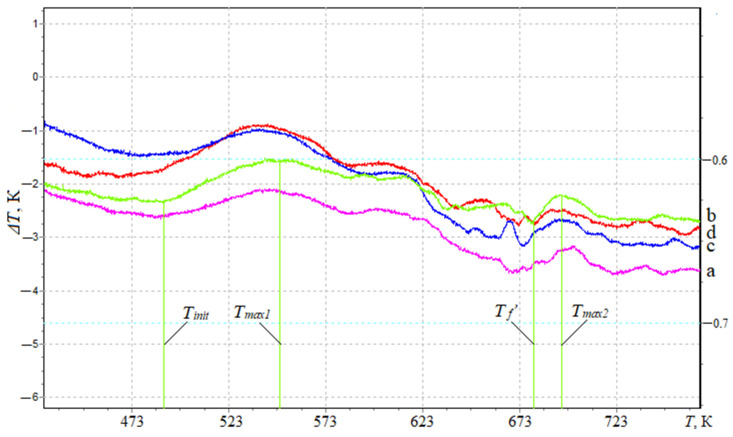
Determination of thermal effects by the method of differential thermal analysis of composites filled with nanodispersed condensed carbon: (a) 0.025 pts.wt.; (b) 0.050 pts.wt.; (c) 0.075 pts.wt.; (d) 0.100 pts.wt.

To register thermal effects and determine the ignition temperature of the developed nanocomposites, differential thermal (DTA) analysis is used (Figure 5). It was found that the maximum value of the initial temperature of the exoeffect—T_init_ = 486.8 K—was typical for composites filled with NCC particles with a content of *q* = 0.050 pts.wt. (Table 5).

The initial temperature of the exoeffect corresponds to the beginning of thermal oxidation of the composite, since no mass loss is observed on the TGA curves (Figure 2). At the same time, there is a possibility of a carbonized layer on the surface of the polymer (as a result of oxidation when the polymer is heated), which acts as a heat-insulating layer that limits the access of the oxidant; thereby, suppressing the destruction of the polymer.

At the same time, analyzing the DTA and TGA curves, a loss of polymer mass was observed after the maximum value of the exoeffect (T_max1_), which indicated the release of gaseous products due to the increase in temperature and the loss of the heat-insulating carbonized layer.

Based on the analysis of DTA curves, two peaks with characteristic maxima for filled composites are established (Table 5, Figure 5). It is believed that T_max1_ corresponds to the temperature at which increased mobility and deformation of bonds occurs due to the structural changes in the nanocomposite, while the oxidation process was replaced by dissociation processes— the chemical and physical decomposition of the oxidized component. Meanwhile, T_max2_ corresponds to the oxidation temperature of the nanoadditive, which in order of magnitude coincides with the characteristics of the filler (the oxidation temperature of the nanofiller is 583–623 K). Thus, the thermal effects of T_max1_ will be greater than T_max2_ in absolute value and the time parameters of the course of the thermal reaction. At the same time, for the quantitative analysis and practical application of the developed polymer materials, the most informative metrics are the initial temperature of the exoeffect (T_init_) and the maximum value of the exoeffect (T_max1_), at which the structural changes in the composites occur. At the same time, the final temperature of the exoeffect (T_f_’) is also important, since in this temperature range, the activation energy is determined by a mathematical method, which makes it possible to estimate the degree of crosslinking of the polymer. Analyzing the final temperature of the exoeffect, an increase in the activation energy is established from E_a_ = 138.8 kJ/mol (*q* = 0.025 pts.wt.) to E_a_ = 170.1 kJ/mol (*q* = 0.050 pts.wt.), which indicates the stability of physical–chemical bonds in relation to destruction under the influence of temperature due to the arrangement of the structure of the polymer. Then, the introduction of nanofiller into the polymer at a content of *q* = 0.075–0.100 pts.wt. ensures a decrease in the value of the activation energy, which can be caused by agglomeration of the additive in the volume of the polymer.

### 3.4. IR Spectral Analysis of Composites Filled with a Nanodispersed Condensed Carbon

**Figure 6:**
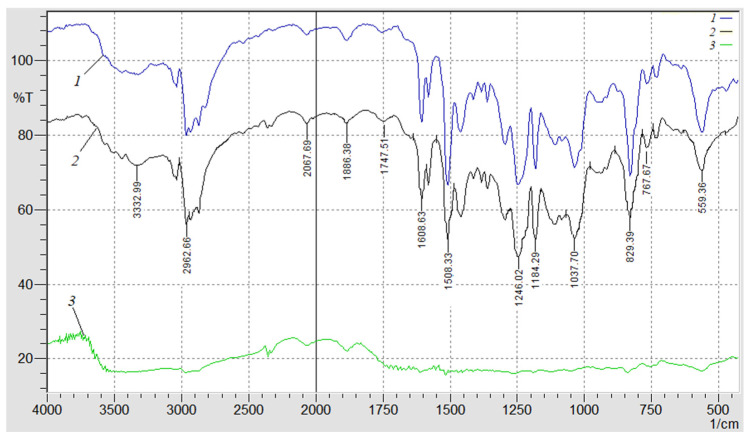
IR spectral analysis of the polymers (0.050 pts.wt.): 1—control sample spectrum; 2—NCC spectrum at the initial temperature of the exoeffect; 3—NCC spectrum at the maximum value of the exoeffect.

In order to confirm the above provisions and specify the permissible temperature range at which it is possible to use the developed nanocomposites, an IR spectral analysis was additionally performed (Figure 6). The registration and analysis of the IR spectra were carried out in stages, in order of increasing temperature (Figure 5). First, the IR spectrum of the composite with the optimal content of nanodispersed condensed carbon, which was not exposed to the influence of temperature (Figure 6, spectrum 1), was obtained. That is, it is the IR spectrum of the control sample to which others are compared that is exposed to the influence of temperature. Then, the IR spectrum of the composite filled with NCC is recorded with a content of *q* = 0.050 pts.wt. (Figure 6, spectrum 2) at the initial temperature of the exoeffect T_init_ = 486 K (Figure 5).

A decrease in the relative value of the peak area from *S* = 18.9% to *S* = 16.1% at the wave number *ν* = 559 cm^−1^ was observed (Figure 6, spectrum 2). This indicates the deformation fluctuations of the aliphatic chain -C-H- with increasing temperature. In addition, a decrease in the relative value of the peak area in the range of wave numbers *ν* = 829–1246 cm^−1^ was also observed, which indicates the mobility and deformation of the macromolecule segments of the ED-20 epoxy oligomer and C-C bonds of the aromatic ring. A decrease in the relative value of the peak area from *S* = 14.4% to *S* = 12.3% at *ν* = 1508 cm^−1^ indicates the increased mobility and deformation of the amine groups of the polymer. The analysis of the IR spectrum in the range of wave numbers *ν* = 1608–3332 cm^−1^ allows for it to be stated that there are no significant structural changes in the polymer.

In the future, the IR spectrum of the composite filled with NCC is recorded (Figure 6, spectrum 3) at the temperature T_max1_ = 553.3 K (Figure 5). A further decrease in the relative value of the peak area from *S* = 16.1% (Figure 6, spectrum 3) to *S* = 2.1% (Figure 6, spectrum 3) at the wave number *ν* = 559 cm^−1^ was observed, which indicates the destruction of the aliphatic chain -C-H-. In the *ν* = 767–1747 cm^−1^, the absence of peaks was observed, which indicates the destruction of a significant number of epoxy amine, C-C-, -C-H- groups. At wave numbers ν = 1886–3332 cm^−1^, no destruction of physical and chemical bonds was detected, only a decrease in the relative size of the peak area was observed. This indicates the resistance of C=O, C-H, O-H groups of the polymer to the influence of the above-mentioned temperature. Therefore, the decrease in the relative size of the peak area and their absence at the wave numbers *ν* = 767–1747 cm^−1^ (at T_max1_) confirms the assumption made earlier about the mobility and deformation of the bonds due to dissociation of the nanocomposite.

It should be noted that (T_max2_) was not given in this study, since the use of the developed materials in these temperature ranges was not relevant due to the destruction of a significant number of chemical bonds.

## 4. Conclusions

The results of studies of the effect of nanodispersed condensed carbon on the course of thermal transformations of polymer composite materials allow the following conclusions to be drawn. The change in the mass of polymer materials in the temperature range Δ*T* = 303–873 K is determined by the method of thermogravimetric analysis, which made it possible to assess the stability of their physical and chemical bonds under the influence of a thermal field. It is proven that composites with a nanofiller content of *q* = 0.050 pts.wt. are characterized by the lowest initial mass loss temperature T_0_ = 624 K. The stability of the physico-chemical bonds to the influence of temperature of the developed nanocomposites is determined by the Broido method. It was found that nanocomposites containing *q* = 0.050 pts.wt. were characterized by the highest heat resistance of nanodispersed condensed carbon, since the maximum value of the activation energy (among the studied nanocomposites) was E_a_ = 170.1 kJ/mol. On the basis of the differential thermal and IR spectral analysis of the developed nanocomposites, it was proven that no absorption bands were found in the temperature range ∆*T* = 303–486 K. That is, the absence of structural transformations (destruction of chemical bonds) made it possible to use the developed materials up to the maximum temperature T = 486 K. The behavior of the developed materials at temperatures T_max1_ = 553 K (ignition temperature) and T ˂ 553 K deserves special attention. It was not possible to use such materials, since the destruction of a large number of bonds of the nanocomposite was observed.

We have developed a new, simplified technology for the formation of epoxy materials (Section 2). The advantage of the proposed technology over existing solutions is a simplified forming technology, which provides for a reduction in costs for the production of new materials; it allows materials to be formed in the conditions of the ship, making it possible to transport and store such materials on the ship on the one hand and to ensure high operational characteristics (compared to analogues) on the other hand. A new approach to determining the maximum temperature at which it is possible to use the new materials without changing their properties is presented. It consists of a combination of modern research methods (DTA, IR spectral analysis) and mathematical methods to research the exothermic reactions that occur when polymer materials are heated.

## Figures and Tables

**Table 1:** Structural changes in polymers under the influence of temperature.

Content of a Nanodispersed Condensed Carbon, %	T_0_, K	T_5_, K	T_10_, K	T_20_, K	T_f_, K	ε_m_, %
0.025	618.0	619.8	633.3	646,6	714.0	63.3
0.050	624.9	628.5	634.6	648.1	718.7	60.3
0.075	556.3	616.5	629.2	641.7	714.0	69.3
0.100	610.0	620.3	630.9	642.8	712.4	70.0

**Table 2:** Change in the polymers mass according to the results of TGA analysis.

*T*, K	Sample Mass (100 − Δm), %			
	Content of a Nanodispersed Condensed Carbon, *q*, wt.%			
	0.025	0.050	0.075	0.100
573	−12.33	−18.62	2.07	−6.21
583	−12.33	−17.93	2.41	−4.83
593	−10.67	−17.24	3.10	−3.10
603	−9.00	−14.48	6.21	−1.72
613	−5.33	−11.38	10.00	1.38
623	2.67	−2.07	16.21	8.62
633	14.00	7.24	25.52	18.62
643	22.67	16.21	35.17	27.59
653	29.67	24.48	44.14	35.86
663	36.67	31.72	54.14	45.86
673	45.33	40.00	62.76	53.10
683	52.33	47.24	70.00	60.00
693	56.67	52.07	75.52	65.86
703	61.33	55.86	82.76	70.00
713	63.00	58.28	84.83	72.07

**Table 3:** Calculated logarithmic dependence of mass on the reciprocal temperature.

*T*, K	ln{ln[100/(100 − Δm)]}			
	Content of a Nanodispersed Condensed Carbon, *q*, wt.%			
	0.025	0.050	0.075	0.100
573	–	–	−3.868	–
583	–	–	−3.712	–
593	–	–	−3.457	–
603	–	–	−2.748	–
613	–	–	−2.250	−4.277
623	−3.611	–	−1.733	−2.406
633	−1.892	−2.588	−1.222	−1.580
643	−1.359	−1.733	−0.836	−1.131
653	−1.044	−1.270	−0.541	−0.812
663	−0.784	−0.963	−0.249	−0.488
673	−0.504	−0.672	−0.012	−0.278
683	−0.300	−0.447	0.186	−0.087
693	−0.179	−0.307	0.342	0.072
703	−0.051	−0.201	0.564	0.186
713	−0.006	−0.135	0.634	0.243

**Table 4:** Activation energy of polymer materials filled with a nanodispersed condensed carbon.

Content of a Nanodispersed Condensed Carbon, *q*, wt.%	*X_HD_*	*X_k_*	*X_i_*	*Y_H_*	*Y_k_*	*Y_i_*	*tq_(φ)_*	Energy Activation E_a_, kJ/mol
0.025	1.605	1.403	0.202	−9.230	−12.605	3.375	16.706	138.8
0.050	1.580	1.403	0.177	−13.286	−16.908	3.622	20.464	170.1
0.075	1.745	1.403	0.342	−4.863	−11.741	6.878	20.112	167.2
0.100	1.631	1.403	0.228	−6.5703	−11.060	4.490	19.693	163.7

Note: *X_HD_*—coordinates of the length of the line along the abscissa axis (start); *X_k_*—coordinates of the length of the line along the abscissa axis (end); *X_i_*—the length of the line along the abscissa axis; *Y_H_*—coordinates of the length of the line along the ordinate axis (start); *Y_k_*—coordinates of the length of the line along the abscissa axis (end); *Y_i_*—the length of the line along the abscissa axis; *tq_(φ)_*—the tangent of the slope angle φ of the logarithmic dependence.

**Table 5:** Structural change in polymers according to the results of DTA analysis.

Content of a Nanodispersed Condensed Carbon, *q*, wt.%	Temperature Intervals of Exoeffects				Maximal Temperature Exoeffects, T_max_, K	
	T_init_, K	T_f_’, K	∆T_1_, K	∆T_2_, K		
					Peak1/T_max1_	Peak 2/T_max2_
0.025	485.5	666.3	184.8	1.50	545.5	683
0.050	486.8	676.3	193.5	0.73	553.3	692
0.075	471.7	670.6	198.9	2.18	540.1	674
0.100	460.0	670.5	210.5	2.39	542.2	680

Note: *T_max_*_1_—exoeffect maximum value composites; *T_max_*_2_—the oxidation temperature of the nanoadditive.

## Data Availability

Data are contained within the article.
